# The nexus between HRM, employee engagement and organizational performance of federal public service organizations in Ethiopia

**DOI:** 10.1016/j.heliyon.2020.e04094

**Published:** 2020-06-15

**Authors:** Assefa Tsegay Tensay, Manjit Singh

**Affiliations:** a Department of Management, University of Gondar, Ethiopia; b School of Applied Management, Panjabi University, Patiala, India

**Keywords:** Organization performance, Public service organizations., Employee engagement, Human resource management, HRM-Performance nexus, Management, Business management, Business

## Abstract

The study of HRM and Performance of public service is an extremely relevant theme. Although studies on the HRM-performance link have been well documented, the results were inconclusive. Besides, previous studies have paid little attention to the public service from developing country's context. Drawing on the AMO Model and SET, the present study examined the effect of HRM System on Employee Engagement and Organizational Performance. Likewise, this study tested the intervening effect of Employee Engagement in the HRM-performance link. Using a sample of federal employees (n=340) in government organizations, we tested the hypothesized three-factor model using SEM. The finding of the study shows that there is a positive and significant relationship between HRM, Employee Engagement and Organization Performance. Besides, employee engagement partially mediated the link between HRM and Performance. Concerning the effect of the individual HR practices, the present study revealed a positive and differing effect of HR practices on both engagement and performance although the magnitude effect is smaller than, the combined effect of the HR practices together. Moreover, Autonomy was identified as an important driver of both engagement and performance. This result contributes to the HRM-performance debate. As a final point, the present study incorporates conclusions, implication and future research direction.

## 1 Introduction

It is widely recognized that the performance of public sector organizations has become an increasingly critical issue in this knowledge-based economy (Vermeeren, 2014). Public service organizations are government-owned and funded organs that provide basic services to its citizens (Obiageli et al., 2016*;* Knies et al., 2017*).* In connection with this*,* Knies and colleagues (2018) argued that basically, the welfare of the country depends on the performance of the public service organizations. To realize an eloquent service delivery in the public service, proper management of the Human Resource (hereinafter: HR) should materialize (Tessema and Soeters, 2006). Armstrong and Taylor (2014) emphasized that HR is a basic input that contributes enormously to the performance of organizations. Similarly, Tessema and Soeters (2006) discussed that HR practices are essential to enhance quality of services offered by the government. The basic issue here is that HRM is a critical function in organizations that influences their performance (Macky and Boxall, 2007). Without HR, organizations are non-living creature and it is believed that it is considered as a source of comparative advantage (Armstrong and Taylor, 2014). Thus, the study of HRM and Performance of public service is an extremely relevant theme (Knies et al., 2017).

The HRM and performance relationship has been the critical area of study in the HRM research (Jackson et al., 2014), owing to its potential impact on the functioning of organizations, which assists them to compete and survive in the present-day complex business environment (Darwish, 2013). The author, thus, posited that this conviction has led to research focusing on the effect of HRM on Organizational Performance, which is called HRM-Performance Linkage. Darwish (2013) portrayed that there are two kinds of research streams, created from the idea of the HRM and Performance debate has been happening. With the first research stream, it was assumed that individual/or system of HR practices lead to a direct effect on the performance of organizations (Arthur, 1994; Huselid, 1995; Delaney and Huselid, 1996; Singh, 2004; Alusa and Kariuki, 2015) whereas the second path of research study argued that HR practices may not have a direct effect on organizational effectiveness, suggesting that there is an indirect effect through employee outcomes (Wright and Gardner, 2003; Boselie et al., 2005; Tessema and Soeters, 2006; Kauto, 2008; Huselid and Becker, 2011). The second path of research is called the black-box issue in HRM-Performance research. There are research calls for scholars to conduct studies on the black-box to find an effective mechanism on how HRM affects organizational performance (Wright and Moynihan, 2003*;* Boselie et al., 2005*;* Backer and Huselid, 2006*;* Kauto, 2008*;* Paauwe, 2009*;* Darwish, 2013). To this end, the present study follows the second research stream in the HRM-performance equation. In the ‘black-box’ paradigm, it is not clear how many boxes to be included to link. For example, Savaneviciene and Stankeviciute (2012) posited that “putting too many boxes and items in the model will neither open the black box nor make the model more insightful”. Therefore, using a relevant mediator in the link could weight more.

As it is outlined in the literature, several writers supported those employee outcomes can mediate the link between HRM and Organizational Performance (Messersmith et al., 2011; Zhang and Morris, 2014; Ko and Walter-Smith, 2013; Vermeeren, 2014; Ahmed et al., 2016). However, the results have yielded mixed results (Kehoe and Wright, 2013; Sun et al., 2007; Kuvaas, 2008). Thus, the present study considered employee engagement as a mediator in the abovementioned link because employee engagement is considered as the key employee outcome variable (Robinson et al., 2004; Truss et al., 2013; Witemeyer, 2013; Ruzic, 2015; Patterson et al., 2010) which leads to improved performance outcomes (Sundaray, 2011; Ogbonnaya and Valizade, 2016). In their study, Rich et al. (2010) discovered that job satisfaction, commitment, and job involvement, as a mediating variable, didn't exceed employee engagement in explaining the relationship between the antecedents' factors and organizational performance outcomes. It was established in the literature that job satisfaction, commitment, job involvement, and OCB are highly studied employee outcomes in the field of HRM (Ko and Walter-Smith, 2013) and there is a trend to search for another mediator variable. Likewise, the concept of employee engagement is much broader and stronger than the other attitudinal and behavioral variables (Robinson et al., 2004; Macey and Schneider, 2008; Markos and Sridevi, 2010; Witemeyer, 2013), indicating that job satisfaction, organizational commitment and job involvement are part of employee engagement, but not sufficient for employee engagement and they never replace an employee engagement (Robinson et al., 2004). Moreover, other scholars (Truss et al., 2013; Alfes et al., 2013) call for future researchers to include employee engagement as a mediator between HRM and organizational performance in the public sector context. In any case, eye-catching researchers identify that HRM– employee engagement - organizational performance equation is misty and needs advance examination (see: Guest, 2011*;* Arrowsmith and Parker, 2013*;* Truss et al., 2013). By this rationale, this study selected employee engagement as a mediating variable to fill the ‘black box’ in the HRM-Performance link in public organization from the developing country's context.

Noticeably, the public service sector remains overlooked in HRM-Performance research (Guest et al., 2003; Katou, 2008; Darwish, 2013; Vermeeren, 2014), indicating that the mainstream of strategic HRM studies have relied on empirical evidence from private organizations (Knies et al., 2017). Although several studies investigated the contribution of HRM on the performance of manufacturing and private organizations in the developed countries, empirical evidence was limited in the public service organizations (Boselie et al., 2005; Combs et al., 2006; Gould- Williams; Vermeeren, 2014) and in developing countries like Ethiopia (Singh and Kassa, 2016). On top of this, the results of the foregoing relationship was inconclusive (Guest et al., 2003; Wright and Gardner, 2003; Wall and Wood, 2005; Combs et al., 2006; Guest, 2011) and it is difficult to assume the presence of such a relationship in public service organizations due to the unique nature of public service employees (Combs et al., 2006*;* Vermeeren, 2014*;* Knies et al., 2017*).*

Ethiopia is a very good context to study the HRM-Performance linkage for some reasons. First, it is located in a strategic area in East Africa, owning one of the largest economies in the continent (IMF, 2015). It is widely recognized that public service organizations contribute more to the country's economy (Gould-Williams, 2003; Wright et al., 2005). Unquestionably, the performance of public service organizations is evaluated through the performance of their HR practices (Tessema and Soeters, 2006*;* Kim, 2005). This implies that HRM contributes greatly to improving the performance of public service organizations. Second, the current government of Ethiopia is undertaking various transformations of its public service organizations due to huge pressure from its citizens for better performance (MoCS, 2015). Third, the public service sector is the largest labor-intensive industry in the country, employing more than 1.5 million workers at the regional and federal levels (MoPS & HRD, 2016). Nevertheless, there is a lack of research that examines the contribution of HRM to the performance of public service organization in Ethiopian context (Singh and Kassa, 2016). In light of little study in the area; given the transformation of the Ethiopian economy and more attention of the government towards public service organizations, it is necessary to critically examine the contribution of HRM towards organizational performance through the mediation model.

This study contributes to the HRM-performance debate by providing empirical evidence from developing country's context in the public service through a mediation model. Regarding the organization of the article, the report is divided into five sections. The first section reviews the background of the study. Next, the literature review and Hypothesis formulation were described. Third, we then presented the methodological perspectives employed followed by the analysis and discussion section. Lastly, the implications, limitations and future research directions are included in this study.

## 2 Literature review and hypotheses development

The concept of HRM is one of the fields of management that gained popularity in the last 30 years or so. Although HRM is the most popular research area, there is no universally accepted and generally acknowledged single definition (Paauwe, 2009). For example, Armstrong and Taylor (2014) defined HRM as ‘a strategic, integrated and clear perspective to employment, development, and wellbeing of the person functioning in organizations’. Similarly, Wall and Wood (2005) defined HRM as a term representing an organization's activities of attracting, developing and managing employees'. In the HRM literature, there are different definitions made by several scholars. In their empirical review, Boselie et al. (2005) posited that there is no consensus on the definition and measurement of HRM. The ultimate objective of HRM is to appropriately manage the HR as an asset (Armstrong and Taylor, 2014). Despite the advancement of HRM studies, the subject is still evolving and requires more investigation (Paauwe, 2009). These all implies that the field of HRM is undergoing significant transformation.

In the HRM-performance debate, the conceptualization of the term HRM, and set of practices used are the most basic part (Lepak et al., 2006). The term HRM System is the most critical part because different studies utilized different terminologies and set of practices (Boselie et al., 2005*;* Combs et al., 2006). For instance, Snape and Redman (2010) define HRM system as encompassing of interrelated HR practices. Saridakis et al. (2016) conceptualized HPWP as a set of different but interconnected, mutually reinforcing HR practices. Datta et al. (2005) define it as a system of practices aimed to enhance the skills, commitment, and productivity of employees. In his book, Armstrong (2009) clearly explained that the HRM system as “an integrated and coherent bundle of jointly reinforcing practices”. Also, *Lepak et al. (2006)* classified the HRM system into high-performance work systems, high involvement system, high-commitment systems, the control system of HR, occupational safety and for customer service.

While review of the empirical studies, it was realized that there are various phrases given for HRM System such as a High-Performance Work System (Boxall and Macky, 2009) or High involvement Work Practices (Huselid, 1995) or High-Performance HR practices (Kehoe and Wright, 2013) or High Commitment (Huselid, 1995; Gould-Williams, 2003; Alfes et al., 2013). Nevertheless, they can be a substitution of one other and their ultimate essence is to affect performance outcomes (Wall and Wood, 2005). Scholars in Strategic HRM have advocated that an organization can use HR practices in its system form to drive organizational performance (MacDuffie, 1995*;* Huselid, 1995*;* Boselie et al., 2005*;* Jiang et al., 2012*;* Katou, 2017). In HRM literature, it is suggested that High Commitment HR practices are suitable in service settings (Boxall and Purcell, 2008). To this end, the present study used the term HRM System to refer High Commitment HR Practices, suggesting as a bundle of HRM Practices combined as a coherent system that ultimately affects organizational performance which is consistent with previous studies (Lepak et al., 2006; Vermeeren, 2014; Jiang and Messersmith, 2018).

Regarding the HR practices employed, different researchers applied different sets of practices in their study. According to Savaneviciene and Stankeviciute (2012), there is no commonly acknowledged theoretical rationale for selecting HR practices. Similarly, Recent meta-analysis reviews uncovered that the utilization of HR practices differ dramatically from one study to another (Boselie et al., 2005*;* Subramony, 2009*;* Jiang and Messersmith, 2018). Nonetheless, there is ample commonality as investigates typically shield a significant variety of HR practices (Wall and Wood, 2005). In their empirical review, Boselie et al. (2005) point out four HR practices utilized by several scholars (Recruitment and Selection, Training and Development, Performance Appraisal, Compensation and Reward). In strategic HRM research, Batt (2002) argued that the top four HR practices (Recruitment and Selection, Training and Development, Performance Appraisal, Compensation and Reward) can reflect the major objectives of strategic HRM.

Despite the varying number of HRM practices, Combs et al. (2006) suggested that on average intellectuals can include five to six HR practices in building the HRM System. On the other hand, HR scholars should be cognizant of the research context while selecting HR practices because public services are different from private sectors in terms of objective and HR practices utilization (Knies et al., 2017). The public service is designed to deliver basic services to its customers and the workforce should be capable, motivated and have the opportunity to contribute to meet the goal of the organizations. Some studies (see; Vermeeren, 2014*;* Knies et al., 2017) suggested to include HR practices that encourage public employees’ ability, motivation and opportunity to perform. In review of the literature, opportunity-enhancing practices were missing in the HRM-Performance research (Wood and de Menezes, 2011).

Following prior studies, the present study comprised of six utmost widely used HR practices in the service settings (Recruitment and Selection, Training and Development, Performance Appraisal, Compensation and Reward; Autonomy and Employee Participation). These HR practices are selected using AMO theory (Appelbaum et al., 2000) rather than following random procedure (Boxall and Purcell, 2008; Lepak et al., 2006; Jiang et al., 2012). With the help of AMO model, the present study grouped Recruitment, Selection, Training, and Development as clustered into Ability-enhancing practices; Performance appraisal, compensation, and Reward congregated the motivation-enhancing practice whereas autonomy and employee participation formed the opportunity-enhancing practices and eventually combined all practices to establish the HRM system, which is consistent with previous studies (Subramony, 2009; Jiang et al., 2012; Mostafa, 2013). We assume that if organizations build a fair recruitment and selection procedure, offer impartial training and development opportunity, follow sound performance appraisal system, provide equitable compensation and reward system and allow employees to involve in the process of decision making as well as autonomous in their work, the personnel become more engaged and become productive (Boxall et al., 2007*;* Markos and Sridevi, 2010).

Theoretically, the HRM-Performance relationship is upheld by several theories such as the resource-based approach, AMO framework, Social Exchange theory, behavioral theory, and others (Jiang and Messersmith, 2018). Nevertheless, the present study utilized the two most commonly used theories (AMO and SET). AMO model is one of the commonly used in the nexus between HRM and Performance. The central issue of the model is to build HR system by categorizing the HR practices into ability, motivation, and opportunity-enhancing HR practices that influence performance outcomes through employee reactions (Jiang et al., 2012). *Boxall (2012)* noted that HRM System works through its effect on employee's skill, and knowledge, their readiness to employ effort and their opportunity to their job and organization. Some studies (for example, Sun et al., 2007*;* Messersmith et al., 2011) provide support for social exchange theory to strengthen the nexus between HRM System and Organizational Performance. So, social exchange theory can underpin to understand how HRM System affects organizational performance through employee reaction.

In the theory of performance, Dyers and Reeves (1995) posited that the outcomes of HR are categorized into employee, operational, financial, and marketing outcomes. They argued that HRM first affects the proximal outcomes (HR and operational) which in turn affects the distal outcomes (financial and market). This is because the distance between HRM and Performance is stretched (Paauwe, 2009). Following this concept, different conceptual models were formulated to understand the black-box issues (Paauwe and Richardson, 1997; Boseile et al, 2005; Armstrong and Taylor, 2014). In their review, *Boseile* and colleagues argued that HR practices affect HR, internal, and financial performance respectively. Similarly, Armstrong and Taylor (2014) posited out that HRM first affects employee characteristics such as engagement, commitment, and motivation and if employees have such characteristics then it leads to operational performance such as productivity, quality and customer satisfaction and finally to financial performance.

**Figure 1:**
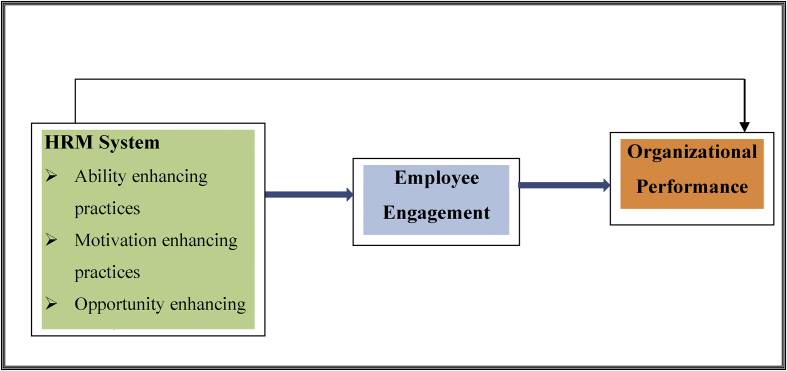
Conceptual model.

Considering the above arguments, the present study build a conceptual model which includes mediation effect in the HRM-performance debate (see Figure 1). As it is illustrated in the figure, the current study proposed that, assuming other things constant, HRM positively and significantly related to employee engagement and organizational performance. In addition, we presumed that systems of HR practices have an indirect effect on the performance of government organizations through the mediation effect of employee engagement. Consequently, the study framed the following hypotheses established on the conceptual model.

### 2.1 Direct relationships

Understanding the nexus between the HRM system and organizational performance has been considered the essential and central research question in strategic HRM (Jackson et al., 2014). Previous studies (Huselid, 1995; Singh, 2004; Gould-Williams, 2003; Boxall, 2012; Alfes et al., 2013; Vermeeren, 2014; Ko and Smith-Walter, 2013; Singh and Kassa, 2016) found that HRM influences both Individual and Organizational Performance. In addition, the findings of the major reviews (Combs et al., 2006; Jiang et al., 2012; Subramony, 2009; Saridakis et al., 2016; Jiang and Messersmith, 2018) confirmed that there is an encouraging relationship between HRM System and Organizational Performance. The results of the different reviews revealed that systems of HR practices have a higher effect on performance outcomes than individual HR practices (Jiang et al., 2012; Subramony, 2009; Saridakis et al., 2016).

Previous studies (Singh, 2004; Amin et al., 2014; Mostafa et al., 2015) found a piece of empirical evidence about the positive relationship between individual HR practices and performance outcomes. For example, recruitment, and selection positively associated with organizational performance (Arthur, 1994; Huselid, 1995; Delaney and Huselid, 1996; Singh, 2004; Katou, 2008; Paauwe, 2009; Amin et al., 2014). Training and Development is another pillar of HRM that enhance organizational performance (Darwish, 2013*;* Armstrong and Taylor, 2014). Similarly, Performance appraisal, Compensation and Reward significantly influence organizational performance (Singh, 2004; Amin et al., 2014). In most of the HRM research studies, employee participation has a positive influence on organizational performance (Singh, 2004; Boxall and Macky, 2009; Katou, 2008; Amin et al., 2014). Moreover, HR scholars (Armstrong, 2009; Kehoe and Wright, 2013; Mostafa et al., 2015) noted that if employees are autonomous in their work assignment, they will improve performance. In light of the above empirical evidence, the researcher postulates the following hypotheses:

Hypothesis 1 *There is a positive and significant relationship between HRM system and organizational performance*

Hypothesis 1A *There is a positive and a significant relationship between the individual HR practices (Recruitment and Selection, Training and Development, Performance Appraisal, Compensation and Reward; Autonomy and Employee Participation) and organizational performance*

Employee engagement is a crucial construct in today's ever-changing and dynamic business environment (Saks, 2006; Rich et al., 2010; Wollard and Shuck, 2011; Alfes et al., 2013). The theoretical background to explain the HRM and Engagement link is the social exchange theory (Saks, 2006), which elaborates why employees involved in the engagement-disengagement continuum. Drawing on the social exchange theory, if organizations invest and treat their employees as a strategic partner and asset, employees become happier and engaged. Few researches empirically examined the relationship between HRM and Employee Engagement (Sundaray, 2011; Alfes et al., 2013; Oliveira and Silva, 2015; Aktar and Pangil, 2017). Overall, the above studies suggested that the use of HRM Practices as a system can positively influence employee engagement. Separately, HR practices also play a role in building an engaged workforce. For example, Saks (2006) argued that the different HR practices such as performance appraisal, reward, and employee participation can enhance employees' engagement level. Robinson et al. (2004) identified training and development, performance appraisal, pay, employee participation, health and safety as a driver of employee engagement. Similarly, Sundaray (2011) posited out that recruitment, training, and performance appraisal and other HR practices are relevant factors that positively affect employee engagement. In their empirical review, Markos and Sridevi (2010) confirmed that HR practices are critical in enhancing the level of employee engagement. Thus, the study proposes the following Hypothesis:

Hypothesis 2 *There is a positive and significant relationship between HRM and employee engagement.*

Hypothesis 2A *There is a positive and a significant relationship between the individual HR practices (Recruitment and Selection, Training and Development, Performance Appraisal, Compensation and Reward; Autonomy and Employee Participation) and employee engagement.*

The ultimate essence of an employee being engaged in the workplace is to enhance Organizational Performance (Rich et al., 2010; Sundaray, 2011; Singh and Karki, 2015; Ogbonnaya and Valizade, 2016). Recently, the issue of engagement is popular in the HRM research, showing that it is critically significant for modern organizations to survive and become successful. In the employee engagement literature, some scholars found that engagement is related to individual outcomes, productivity and organizational performance such as OCB (Alfes et al., 2013; Saks, 2006); organizational effectiveness (Sundaray, 2011) and organizational performance (Harter et al., 2002; Markos and Sridevi, 2010; Singh and Karki, 2015; Ogbonnaya and Valizade, 2016). So, the findings showed that there is a substantial and a positive connection between engagement and organizational outcomes. Thus, we developed a Hypothesis as follows:

Hypothesis 3 *There is a positive and significant relationship between employee engagement and organizational performance.*

### 2.2 Mediation effect of employee engagement

In the HRM-Performance research, a substantial body of studies has examined the nexus between HRM and performance (Singh, 2004*;* Wright and Gardner, 2003*;* Kehoe and Wright, 2013*;* Paauwe, 2009*;* Boselie et al., 2005*;* Guest, 2011*;* Ko and Smith-Walter, 2013*;* Vermeeren, 2014*;* Brito and Oliveria, 2016). Although the range of empirical studies confirmed the positive and significant effect of HRM on Organizational Performance, some scholars (see. Guest et al., 2003*;* Wright and Gardner, 2003*;* Wall and Wood, 2005*;* Combs et al., 2006*;* Guest, 2011*;* Riaz, 2015*;* Brito and Oliveira, 2016) argued that link has mixed results even weaker relationships (Paauwe, 2009). These mixed findings drive researchers to uncover other ways and they believed that there is a “Black Box**”** in the linkage (see; Wright and Gardner, 2003*;* Boselie et al., 2005*;* Katou, 2008*;* Darwish, 2013).

The current trend in the literature is to consider intermediary outcomes in the relationship between HRM-performance equations. Previous HR scholars (Becker and Wright, 2006) noted that the HR practices do indirectly result in performance through changes in the attitudes and behaviors of the workforce. Following this suggestion, the present study takes the Paauwe and Richardson (1997) as an underpinning in examining the effect of HRM System on Public Service Performance using AMO model (Savaneviciene and Stankeviciute, 2012*;* Boselie et al., 2005*;* Lepak et al., 2006) and social exchange theory (Sun et al., 2007; Alfes et al., 2013) to explain the intervening role of employee outcomes (Employee Engagement) in the link.

In the conceptual and empirical studies, employee engagement is considered as employee outcome (Rich et al., 2010*;* Patterson et al., 2010*;* Sundaray, 2011*;* Ahmed et al., 2016*;* Ruzic, 2015). Consistent with previous studies, the current research considered Employee Engagement as HR outcome and hypothesized the mediation role in the connection between HRM and organizational performance. In the literature, there are few studies that support the role of employee engagement as a mediator in the relationship between HRM and performance (Sundaray, 2011; Alfes et al., 2013; Truss et al., 2013; Muduli et al., 2016*)*. In 2006, Saks confirmed the role played by employee engagement as a mediating variable in the bond between antecedents and its consequences. Rich et al. (2010) proved that employee engagement plays as a mediating variable between antecedents and their consequences. Sundaray (2011) confirmed that employee engagement can mediate between antecedents and organizational outcomes. In the UK context, Alfes et al. (2013) confirmed that employee engagement can be an intervening variable in the connection between HRM and performance outcomes. Besides, Truss et al. (2013) concluded that employee engagement can play an intervening role in the link between HRM-Performance. Ruzic (2015) proved the mediation role in the relationship between HRM and organizational performance. Moreover, Muduli et al. (2016) found employee engagement as an intervening variable in the HRM an organizational performance relationship in the service sector. The above analysis and argument highlights the presence of a relationship between HRM, Engagement and outcomes. Following the above arguments, the present study proposed that:

Hypothesis 4 *Employee engagement can mediate the relationship between HRM and organizational performance in Ethiopian public service*.

## 3 Materials and methods

### 3.1 Research setting and sampling procedure

The study was conducted on federal public service organizations. All the federal public service offices operating in Ethiopia are headquartered in Addis Ababa. The city is the hub of Africa where many organizations such as federal offices, diplomats, foreigners, and NGOs are concentrated at. In the Ethiopian context, the public service organizations are structured into the federal, regional and local levels. We focused on the federal level public service organizations for the subsequent justifications. First, the federal public service organizations have a macro-level impact on the social, economic and political activities of the nation. Second, the government gave much attention to public service organizations at the federal level as per the Growth and Transformational Plan of the Country. Third, they are the backbones of the economy and basic sectors for social and economic developments to achieve the country's vision. Fourth, the federal public service offices are composed of a large number of diversified employees from different races, religions, and cultural background which is believed to be representative of the entire country. Finally, the federal public service organizations were chosen for analysis specifically they are better performers and have better HR composition compared to the regional and local organizations. Taken together, the present study emphasized on the federal public service organizations.

In this study, a multi-stage random sampling procedure was engaged by bearing in mind the type of sector as strata. First, the federal public service organizations were categorized into four sectors (strata) and then two organizations were randomly selected from each sector (8 organizations) and Ministry of Public Service and Human Resource Development, the governing body of the country's civil service administration is intentionally included in the sample. The random selection of the sample organizations were done through this process: first, the researcher numbered all the public service organizations in each category on paper slip then mixed these slips; second, the researcher chooses one slip at a time to reach a reasonable sample of nine federal public service organizations for this study. This study took 30% of the total organizations, showing a fair representation of the population organization as the previous studies in public service took 20% of population organizations (see: Gould-Williams, 2003). In the second phase, simple random sampling is used for selecting the employees from the sample organizations from the different departments and sections through the help of the HRM Directorate Office. The HR Director was personally contacted through the official letter and discussed the objective of the research. For the purpose of having a representative sample, the researcher together with the focal person equally draw respondents from different departments and sections, which is grounded on the list of employees.

In SEM analysis, sample size determination is critical and it is noted that large sample size is necessary to improve statistical power and trustworthiness of the results (Kline, 2011). And also *Hair et al. (2010)* asserted that the minimum sample size for a particular SEM model depends on the complexity of the model, the value of communality and other factors. It is argued that the minimum sample size for the SEM model is 200 (Kline, 2016). On the other hand, in multivariate inquiry, Sekaran and Bougie (2013) noted that sample size is proposed as ten times the number of constructs in the research study. Following the suggestions of (Hair et al., 2010*;* Sekaran and Bougie, 2013*;* Kline, 2011*,* 2016), the present study took a sample of 400, which is beyond the minimum sample requirement. To that end, we distributed (n=400) questionnaires in the official language (Amharic) of the country to permanent employees of the randomly selected nine federal public organizations. As a result, we used 340 properly filled questionnaires for final data analysis and the effective rate of return was 85%, which is considered to be sufficient enough to run the SEM analysis.

### 3.2 Measurement of variables and instrument

To test the Hypothesis, *Sekaran, and Bougie (2013*) argued that the constructs under study should be measured. The current study utilized a validated and developed standard questionnaire from previous studies conducted in the public sector was used to measure all the study variables. Responses to all questionnaire items were given on a five-point Likert scale in which 1 = “Strongly disagree” and 5 = “Strongly agree”. As the official language of Ethiopia is Amharic, the questionnaires were administered in Amharic which was originally designed in English, and then interpreted into Amharic through the help of university professors and professionals following the recommendation of research literature. The back-interpreted version was compared with the original to ensure accuracy and consistency of meaning and didn't see any validity problem. Objectively, a pilot test was conducted by randomly selecting employees (n=50) from three sampled and non-sampled public service organizations. The findings of the pilot study demonstrated the high reliability of the instrument. Thus, the result of the pilot study ensured the appropriateness of the measurement instruments in terms of reliability and data collection procedures. The final measurement scale was established after some minor modifications and wordings from the pilot test and actual data was collected from October to December 2017. So, the measurement instruments of the study variables are described below.

#### 3.2.1 Human Resource Management

This index is measured using 31 items, six High Commitment based HR Practices namely: Recruitment and Selection, Training and Development; Performance Appraisal; Compensation and Reward; Autonomy and Employee Participation based on the work developed by *Vermeeren (2014)*. The measurement items were drawn from well-known scholars in the Human Resource Management studies (Ahmad and Schroeder, 2003; Appelbaum et al., 2000; Gould-Williams, 2003; Huselid, 1995; Wright et al., 2005). The items uncover the three AMO model dimensions of HR practices: Ability enhancing practices (Recruitment, Selection, Training and Development), motivation enhancing practices (Performance Appraisal, Compensation, and Reward) and opportunity enhancing practices (Autonomy and Employee Participation) and ultimately form the HRM System by combining scores of each item of all the HR Practices. Commitment based HR practices are critically important in encouraging employees to take more responsibility and ultimately benefits their organization (Combs et al., 2006). By this logic, the present study adopted Vermeeren's High commitment HR practices measurement scale. Although the study was based on standardized measures for each construct, it is advisable to conduct Exploratory Factor Analysis (EFA) to explore the factorability of the indicators due to the circumstantial differences (Gurbuz and Mert, 2011). To test the applicability of the questionnaires in the Ethiopian contexts, the study employed factor analysis using principal component extraction with varimax rotation for HRM. The EFA result revealed that the 31 items are categorized into six factors, which are consistent with the original measurement scale.

#### 3.2.2 Employee Engagement

Typical different scholars utilized diverse estimation scales, proposing no reliably acknowledged tool to assess engagement. In the engagement literature, there are different measurement scales in the measurement of engagement such as Gallup 12-items Worker Engagement Index; the Utrecht Work Engagement Scale (Schaufeli et al., 2006); Job Engagement scale developed (Rich et al., 2010), to mention some. The Utrecht employee engagement scale was widely used in several studies (Christian et al., 2011; Rich et al, 2010; Kulikowski, 2017) and most acknowledged instrument with strong legacy in the literature (Witemeyer, 2013; Kulikowski, 2017) as a result of its massive acceptability in a large number of countries around the world (Bakker and Schaufeli, 2008). To this end, the present study employed the short version (9 items) Utrecht Work Engagement Scale (Schaufeli et al., 2006) as a measurement instrument. In *2017, Kulikowski* reviewed several studies and found that the short version of the Utrecht Work Engagement Scale is the most popular, reliable, and valid and have higher psychometric properties than the 17 item version. To test the applicability of the questionnaires in the Ethiopian contexts, the study employed factor analysis method using principal component extraction with varimax rotation for the short version of the Utrecht Work Engagement Scale and the EFA analysis using principal axis factoring method result discovered that the nine items are categorized into one factor, which is consistent with previous studies (Schaufeli et al., 2006; Shimazu et al, 2008; Kazimoto, 2016).

#### 3.2.3 organizational performance

The Perceived Organizational Performance is measured using 12 items representing various aspects of organizational performance, such as productivity, quality, goal attainment, and customer satisfaction. The measurement scale was adopted from *Kim (2005)*, which was originally developed by *Brewer and Selden (2000)*. A year later, this measurement scale was revised by *Park et al. (2001)*. In the performance literature, some other scholars (for example, Sahin, 2010*;* Kula and Sahin, 2016*;* Emhan et al., 2016) were used in their studies to measure organizational performance. In order to test the applicability of the contextual differences, the present study conducted EFA using the principal axis factoring method with varimax rotation. The EFA result revealed that the twelve items are categorized into one factor, which is consistent with previous studies (Sahin, 2010*;* Kula and Sahin, 2016*;* Emhan et al., 2016).

#### 3.2.4 Control variables

In order to see the true relationship between HRM and Performance, we considered the demographic variables as control variables (Boseile et al, 2005*;* Kim, 2005). The demographic characteristics in the survey are coded in this way:, gender (1 = male, 2 = female); age (1 = less than 25, 2 = 25–30 years, 3 = 31–40 years, 4 = 41–50 years and 5 = greater than 50 years); education (1 = high school, 2 = certificate/diploma, 3 = Bachelor degree, 4 = Master degree and 5 = PhD Degree) and length of service (1 = less than 1, 2 = 1–5 years, 3 = 6–10 years, 4 = 11–20 years and 5 = greater than 20 years) and (1 = professional and scientific service, 2 = administrative service, 3 = sub professional service, and 4 = clerical and fiscal service). The assumption of the control variable is that different groups of employees within the study organizations may be managed and treated differently. To test for control variables among the demographic features, one-way ANOVA was accomplished. The ANOVA result analysis established no difference of response across gender, age, education, experience, institutions or job grade, indicating that all the demographic variables have an insignificant effect on organizational performance, which is consistent with previous studies (Sahin, 2010; Singh and Karki, 2015; Brito and Oliveira, 2016). The reason could be the fact that civil service employees were managed with the standardized procedures in Ethiopian context. In addition, the majority of the employees have a small portion of the difference in education level, and job grade. Moreover, it was revealed that most of the employees were categorized as productive age (30–40). Hence, the present study reports control free (Field, 2009), which is consistent with previous studies (Fey et al., 2000; Mostafa et al., 2015).

### 3.3 Method of data analysis

This study analyzed the data using both SPSS (version 24) and AMOS (version 23). Before going into further discussion, preliminary analysis to test for missing data, outliers, normality and multicollinearity concerns were performed to improve the accuracy of the data preparation and screening process which is critical stage in SEM analysis (Hair et al., 2010*;* *Kline, 2011**,*^2016^). Then, descriptive statistics and Pearson correlation was performed to evaluate the reliability and association between constructs. Next, CFA by Maximum Likelihood Estimation, as a method, was performed to verify the construct validity and appropriateness of the measurement models. As a final point, Hypothesis testing was accomplished using SEM to examine the direct and indirect effect of HRM on public service performance.

### Ethical approval

The study is done based on perception of public servants as per their willingness through official communication with study organizations. As a social science study, it is free of any human experiment and doesn't demand ethical approval. Thus, the intuition didn't require any ethical approval.

## 4 Results

### 4.1 Characteristics of respondents

The demographic profile data (n=340) result indicated that the majority of the respondents are male, married, holds a bachelor's degrees, matured, experienced and fall under the category of professional science job grade. Specifically, most of the respondents lie on the age categories ranging from 31-40 years (39.4%) followed by the age group of 25–30 (24.7%) and the age group of 41–50 years (20.6%). Of the 340 respondents, 60.3% are male employees. Regarding the education level, many of the respondents have a first degree (56.2%), followed by a second degree (28.8%). With respect to experience, many of the respondents do have a service level between 6-10 years, followed by between 11-20 years. In addition, reasonable numbers of respondents have experience of more than 20 years (20.9%). Of the respondents, 57.6% are married followed by unmarried once (38.2%) whereas respondents with divorced marital status are very insignificant (4.1%). Regarding the type of job category, the majority are categorized under professional science. Thus, the study can be generalized because respondents are representative of the population in terms of gender, age, education, experience and job category which is consistent with the government reports of the country.

### 4.2 Descriptive statistics and correlation results

Table 1 shows the descriptive statistics and correlation results of the study variables. Respondents were requested their opinion concerning the execution of HR practices, their level of engagement and the performance of their organizations. The finding of the descriptive statistics publicized that the mean scores of the study measures were above the midpoint (3.00) of the rating scale, showing that employees perceived positively with respect to the implementation HRM (Mean=3.37;SD=0.519), level of engagement (Mean=3.50;SD=0.953)and performance of organizations (Mean=3.16;SD=0.688).This is an encouraging result for public service organizations. However, there is some deviation among respondents in perceiving the study variables. Concerning internal consistency, it was demonstrated that the Cronbach alpha reliability of the study variables was very good, with all Cronbach's alpha coefficient greater than the threshold, indicating that the data gathered was reliable and can be generalized to the entire population. As it is expected, the correlation coefficient result confirmed the associations between study measures were statistically significant and in the same direction.

**Table 1:** Cronbach's alpha and correlation result analysis.

	Cronbach's Alpha	HRM	EEA	POP
HRM System	**0.92**			
Employee Engagement	**0.96**	.432∗∗		
Perceived Organizational Performance	**0.94**	.538∗∗	.714∗∗	

∗∗ correlation is significant at the 0.01 level (2-tailed).

### 4.3 Preliminary analysis

Before running directly into CFA operation, the present study made a preliminary analysis of the accuracy of the data. It is widely recognized that data preparation and screening are critical issues in SEM for the mere fact that researchers may commit mistake in entering data into computer file; most of the estimation methods require assumptions and data-related problems can make SEM computer tools to produce illogical results (Hair et al., 2010*;* Kline, 2011*,* 2016). Thus, this study examined the missing data using excel and the list-wise deletion method. Thus, no missing data were identified. The Mahalanobis distance rule revealed that there is no problem with outliers. Regarding the normality test, the present study calculated the skewness and kurtosis and it was found that the values are within the normal range, indicating that there is no problem of normality of data. Lastly, the issue of multicollinearity, using the VIF and tolerance test, was examined and found that there is no multicollinearity concern. Appropriately, the current work examined the missing data, outlier, normality and multicollinearity issues and didn't perceive any infringement. In view of that, the current study discovered that data preparation and screening were properly analyzed and the variables are eligible to enter into SEM analysis.

### 4.4 Evaluation of the measurement model

Variance-based Structural Equation Modeling (SEM) technique was employed to analyze both the measurement and structural model respectively (Hair et al., 2010; Henseler et al., 2015). The current study hypothesized three-factor measurement model (Human Resource Management, Employee Engagement and Perceived Organizational Performance) aimed at validating the appropriate fitness of the proposed model. The factoring method result discovered that the nine items of employee engagement and twelve items of organizational performance are categorized into one factor. Similarly, previous studies (Schaufeli et al., 2006; Shimazu et al, 2008; Kazimoto, 2016) argued that employee engagement considered as the first order factor. Concerning organizational performance, prior studies (Sahin, 2010*;* Kula and Sahin, 2016*;* Emhan et al., 2016) discovered that the construct is considered as a one-factor construct. On the other hand, the EFA result of this study and previous studies ((Jiang et al., 2012; Mostafa, 2013; Mostafa et al., 2015; Korff et al., 2017) consider HRM as higher order construct. To this end, the second-order of the HRM construct is built by categorizing the six HR practices into ability, motivation and opportunity-enhancing HR practices based on the AMO theory (Boxall, 2012; Jiang et al., 2012), which is consistent with previous studies (Jiang et al., 2012; Mostafa, 2013; Korff et al., 2017).

To test the CFA of each construct, item parcels were developed for HRM construct due to the presence of a large number of items (31 items). Some scholars (Rogers and Schmitt, 2004; Little et al., 2002) argued that item parceling is appropriate when the objective of the study is to examine the relationship between latent constructs (Little et al., 2002) and there exists a larger number of items (Matsunaga, 2008). Item parceling is a measurement practice that is widely used in latent variable analysis such as exploratory factor analysis and SEM (Little et al., 2002). Rogers and Schmitt (2004) defined parcel as “the arithmetic sum of the indicators allotted to it”. In SEM literature, it was clearly identified that item parceling has many benefits. For example, Matsunaga (2008) pointed out that the benefit of parceling is an enhancement of scale communality, an increase in the common-to-unique ratio for each indicator, and lessening of random error. It also produces higher reliability and yields stable parameter estimation (Little et al., 2002; Rogers and Schmitt, 2004; Hair et al., 2010). Moreover, item parceling improves model efficiency (Matsunaga, 2008).

Following the suggestion of Little et al. (2002)*,* the present study employed internal consistency approach to form item parceling. To this end, HRM has six dimensions and items of each dimensions are parcel together. To enter the parcels into CFA, the present study follows the procedure identified by prior scholars (Matsunaga, 2008*;* Kishton and Widaman, 1994), which advised to conduct EFA and reliability analysis in confirmatory research. The result of EFA and internal reliability analysis are presented in Table 2. As it is illustrated in the table below (see Table 2), the variance explained are greater than 50 % and the internal reliability analysis are greater than 0.7, indicating that the parcels are appropriate to enter into CFA.

**Table 2:** Internal consistency reliability analysis and Explanatory Factor Analysis.

Parcel	Number of items	Cronbach's Alpha	# of extracted components	% of variance explained
Recruitment and Selection	4	0.884	1	74
Training and Development	8	0.910	1	62
Performance Appraisal	6	0.775	1	52
Compensation and Reward	3	0.814	1	69
Autonomy	6	0.865	1	59
Employee Participation	4	0.825	1	66

In SEM analysis, measurement model is the first stage to be analyzed with the objective of testing construct validity (convergent and divergent validity) of the study variables (Hair et al., 2010; Kline, 2011). To assess convergent validity, factor loading, average variance extracted and composite reliability was considered *(*Hair et al., 2010*).* The acclaimed values for factor loading are supposed to be greater than 0.55, for AVE at least 0.5 and that of CR to be greater than 0.7 (Hair et al., 2010; Kline, 2016). Thus, the CFA result of each construct is displayed in Table 3, which shows that the factor loading of each indicator is above the threshold and significantly related to the latent factors at p < 0.001. Besides, the AVE of each first order and second-order constructs were above 0.5 and that of CR is greater than 0.7 except for Ability-enhancing and Opportunity-enhancing HR bundles which are between 0.6 and 0.7. They are still within the acceptable range provided that the other indicators of model's construct validity are good (Hair et al., 2010*;* Bagazzi and Yi, 1988).

**Table 3:** Measurement model.

First order Construct	Second order Construct	Items	Factor Loading	AVE	CR
Ability-Enhancing		RS	0.65	0.516	0.68
		TD	0.78		
Motivation-enhancing		PA	0.86	0.594	0.74
		CR	0.67		
Opportunity-enhancing		AU	0.65	0.516	0.68
		EP	0.78		
	HRM	Ability	0.806	0.6963	0.87
		Motivation	0.870		
		Opportunity	0.826		
Employee Engagement		V1	0.889	0.7650	0.98
		V2	0.851		
		V3	0.850		
		D1	0.884		
		D2	0.865		
		D3	0.870		
		AB1	0.894		
		AB2	0.890		
		AB3	0.877		
Organizational Performance		OP1	0.741	0.5811	0.96
		OP2	0.793		
		OP3	0.696		
		OP4	0.737		
		OP5	0.774		
		OP6	0.703		
		OP7	0.753		
		OP8	0.806		
		OP9	0.721		
		OP10	0.777		
		OP11	0.763		
		OP12	0.867		

Note: Ability = Ability-enhancing HR practices; Motivation = Motivation-enhancing HR practices and Opportunity = Opportunity-enhancing HR practices.

As it is shown in Table 4, finding of the study established that convergent validity was confirmed as the AVE and CR of each construct were beyond the threshold (Hair et al., 2010; Kline, 2016).

**Table 4:** Convergent validity results of the individual constructs.

Construct	AVE	CR	Construct Validity
Human Resource Management System	0.6963	0.87	Confirmed
Employee Engagement	0.7650	0.98	Confirmed
Perceived Organizational Performance	0.5811	0.96	Confirmed

**Figure 2:**
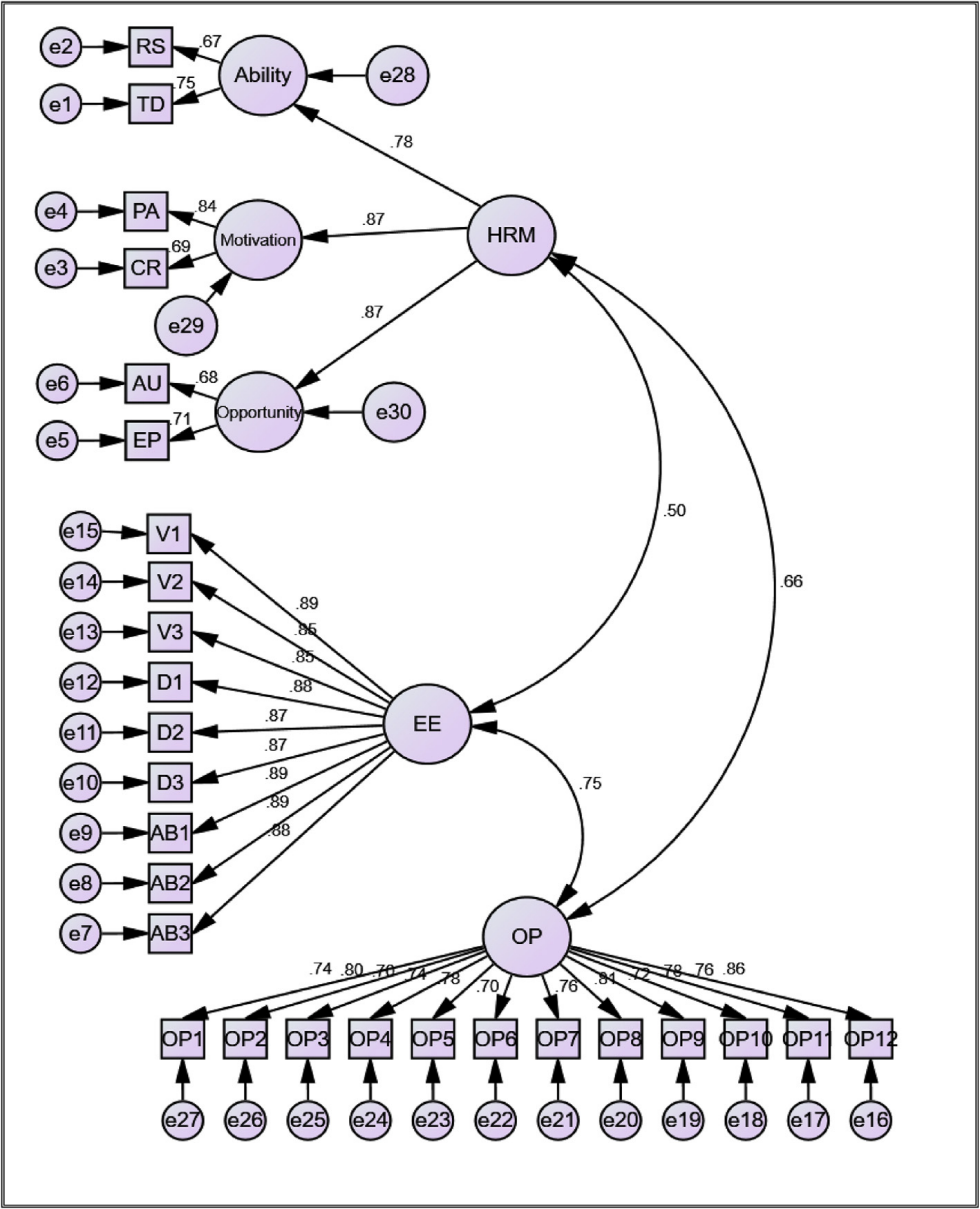
**CFA result for the three-hypothesized measurement model.** Note: Ability; Motivation and Opportunity-enhancing HR practices. HRM = Human Resource Management; EE = Employee Engagement and OP = Organizational Performance. RS = Recruitment and Selection; TD = Training and Development; PA = Performance Appraisal; CR = compensation and Reward; AU Autonomy and EP = Employee Participation.

The second objective of measurement model is to test discriminant validity. Divergent validity is the degree to which a construct is strictly different from others (Hair et al., 2010*;* Henseler et al., 2015). To verify the discriminant validity, overall CFA was conducted by combining the three constructs together (exhibited in Figure 2). The CFA result shows that the overall measurement model was properly fit with the sample data (χ2/df = 1.606; RMR = 0.030; CFI = 0.972; TLI = 0.970; and RMSEA = 0.042), which are consistent with the fit indices of (Hair et al., 2010; Kline, 2016*)*. As shown in Figure 2, the factor loading of each indicator was positively and significantly related to the latent constructs.

To ensure the divergent validity, we followed two mechanisms. One way is just by looking at the correlation between the constructs. It is recognized that the covariance between constructs should be below 0.85 (Field, 2009*;* Awang, 2012). As presented in Figure 2, the correlation between constructs is within the acceptable range (0.504–0.746), which indicates for the support of divergent validity. The other mechanism is by comparing the AVE of each construct with the square of the correlation between constructs (Henseler et al., 2015). Table 5 unveiled that the AVE of each construct is greater than the square of the correlation between constructs. Thus, divergent validity is confirmed, which indicates that the three constructs are different.

**Table 5:** Divergent validity result.

Construct correlations	Correlation result ®	Correlation result r^2^	AVE of both Constructs	Divergent Validity
EE<--> HRM	0.504	0.2540	0.7650 & 0.6963	Established
OP<--> EE	0.746	0.5565	0.5811& 0.7650	Established
OP<--> HRM	0.664	0.4409	0.5811& 0.6963	Established

Note: AVE of both constructs should be greater than **r**^**2**^.

This study also performed a common method bias test. In many social science studies, the issue of common method variance is widely recognized as a major problem (Sekaran and Bougie, 2013). To test for common method bias, we followed the suggestion of (Podsakoff et al., 2012). The present study employed both procedural and statistical techniques. The factor analysis result demonstrated that the total variance explained by one factor was 33.16% which is below the minimum requirement. From an exploratory factor analysis perspective, the result of Harman's one-factor test shows that the current study is free from common bias.

### 4.5 Structural model result

**Figure 3:**
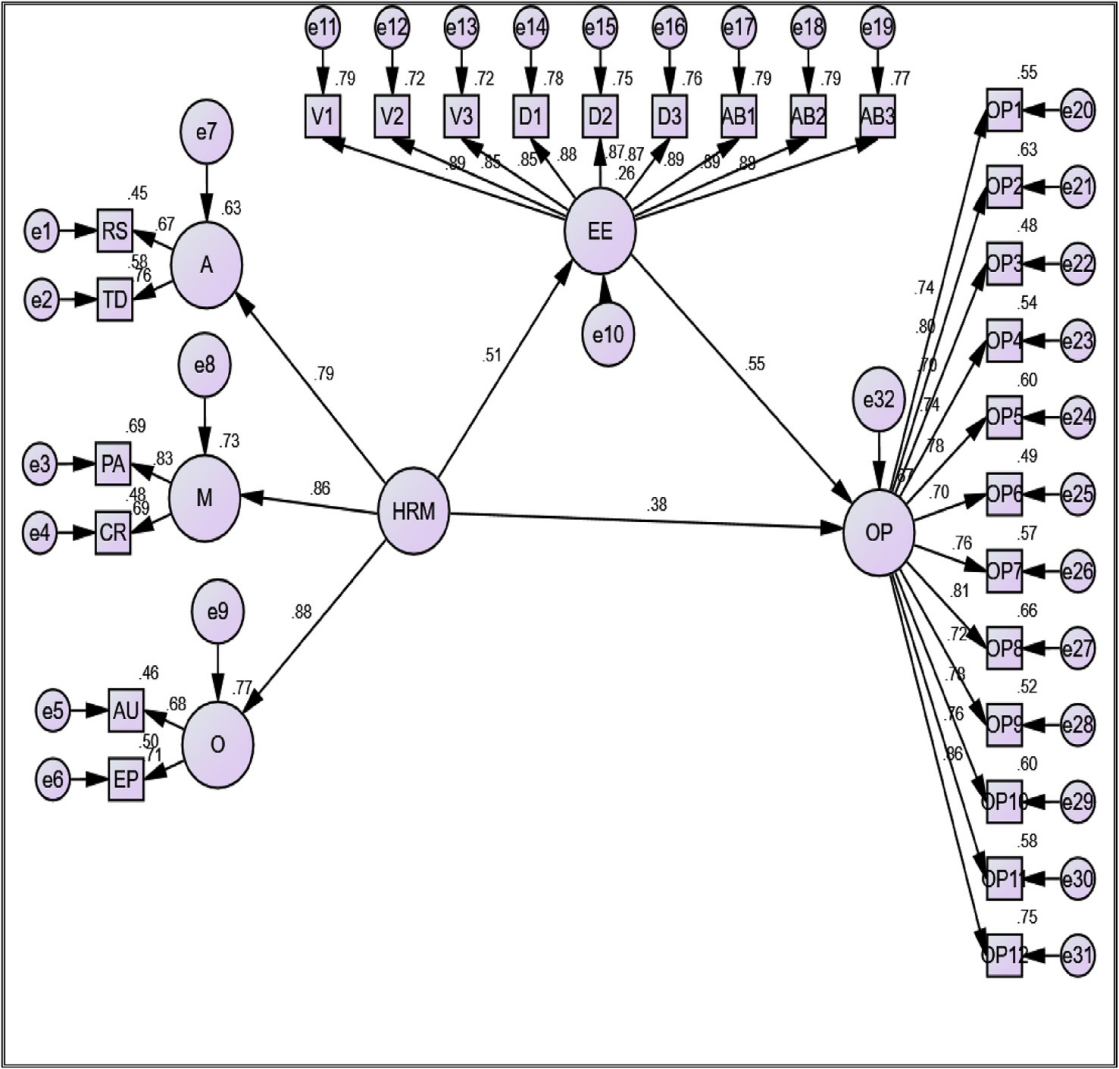
Proposed Structural Model. Note: A = Ability; M = Motivation and 0 = Opportunity-enhancing HR practices. HRM = Human Resource Management; EE = Employee Engagement and OP = Organizational Performance. RS = Recruitment and Selection; TD = Training and Development; PA = Performance Appraisal; CR = compensation and Reward; AU Autonomy and EP = Employee Participation.

The second stage of SEM analysis is evaluating the structural model (Hair et al., 2010*;* Kline, 2011*,* 2016). Preceding to testing the Hypothesis, first the structural model fitness with the theory was validated based on the fit measurement indices. The CFA result has shown that the structural Model demonstrated that the sample data adequately fit with the theory (χ2/df = 1.606; RMR = 0.030; CFI = 0.972; TLI = 0.970; and RMSEA = 0.042). Figure 3 shows the structural proposed model proposing the mediation effect of employee engagement on the linkage between HRM and Organizational Performance. In this model, the path from HRM and Employee Engagement as well as the path from Employee Engagement to organizational performance was found statistically significant and in the same direction, suggesting that the result supports the proposed conceptual model and earlier theoretical arguments. The study also found that all the standardized regression weights between the items and latent constructs were statistically significant at p-value < 0.001. Moreover, HRM System and employee engagement together account for 67% variance in perceived organizational performance, indicating that both HRM and engagement are critically relevant factors in enhancing organizational performance.

### 4.6 Hypothesis testing

To test the Hypothesis of the study, we utilized SEM. Hypothesis 1 proposed that HRM positively and significantly affects organizational performance. As it is displayed in Table 6, the SEM analysis result demonstrated that HRM has a positive and significant effect on organizational performance (ß = .663, p < .001). Thus, the hypothesis one was accepted. Regarding, the effect of the individual HR practices, the result of the study indicated that Recruitment and Selection (ß = .381, p < 0.001); Training and Development (ß = .341, p < 0.001); Performance Appraisal (ß = .455, p < 0.001) Compensation and Reward (ß = .438, p < 0.001); Autonomy (ß = .467, p < 0.001) and Employee Participation (ß = .388, p < 0.001) have a direct and positive effect on perceived organizations performance. Within the individual practices, Autonomy and employee participation have a larger effect size on organizational performance respectively than the other individual HR practices.

**Table 6:** The causal effect of HRM on Employee Engagement and Organizational Performance.

Structural Path	SWR	S.E	C.R	P-Value	Result
OP <-- HRM	0.663	0.127	8.146	**∗∗∗**	Significant
EE <-- HRM	0.515	0.165	6.776	**∗∗∗**	Significant
OP <-- EE	0.746	0.038	13.795	**∗∗∗**	Significant

∗∗∗P-Value<0.001.

Hypothesis 2 postulated the positive and significant relationship between HRM and employee engagement. The direct effect of HRM on employee engagement was significant (ß = .515, p < .001), supports for Hypothesis 2. With respect to the individual HR practices, it was found that Recruitment and Selection (ß = .257, p < 0.001); Training and Development (ß = .288, p < 0.001); Performance Appraisal (ß = .286, p < 0.001); Compensation and Reward (ß = .265, p < 0.001); Autonomy (ß = .399, p < 0.001) and Employee Participation (ß = .386, p < 0.001) have a direct and positive effect on perceived organizational performance. Among the individual practices, Autonomy has a larger effect size on organizational performance as compared to the other individual HR practices. In Hypothesis 3, it was projected the direct effect between employee engagement and organizational performance. Thus, the result of SEM scrutiny established a significant and strong effect on organizational performance (ß = .746, p < .001).

The 4^th^ Hypothesis of the study was to test the intervening effect of employee engagement on the HRM-performance link. In mediation analysis, it was identified that there are several methods employed in exploring the mediation effect (MacKinnon et al., 2010). To that end, the current study employed both the bootstrap procedure and Kenney and Barton test, supporting the idea of (MacKinnon et al., 2010) that using two or more statistical techniques improves the robustness of the mediation results. Interestingly, this study tests the mediation role of Employee Engagement in the HRM-Performance debate using the four pre-conditions recognized by Baron and Kenny (1986). The first condition is the presence of a direct link between the HRM and Organizational Performance, which is achieved. Second, a direct relationship between HRM and Employee Engagement is fulfilled. Third, Employee Engagement should demonstrate a positive relationship with Organizational Performance which is true in the present study and finally, the relationship between HRM and Organizational Performance should significantly reduce upon the addition of the intervening variable in the model (Employee Engagement). By this logic, since the introduction of the mediator (Employee Engagement) reduced the effect of HRM on performance (ß = .385, p < .001 and still it is significant (see Figure 3 above). Thus, Employee Engagement plays a partial mediation role, supporting for the hypothesis.

The second method used in examining the mediation effect of employee engagement in the HRM- Performance debate was bootstrap standard error based test. In the AMOS analysis, the researcher performs 5000 number of bootstrap using Bias-Corrected Confidence interval at 95%. Established on the bootstrap procedure, the coefficient path of the model demonstrated that all the direct and indirect effects are statistically significant as it is displayed in the Table 7. The result supported the partial mediation argument.

**Table 7:** The direct and indirect effect of Human Resource Management System on Perceived Organizational Performance.

Hypothesis	Direct Effect	Indirect Effect	Result
OP<--EE<-- HRM	0.385∗∗∗	0.279∗∗∗	Partial Mediation

∗∗∗P-Value<0.001.

## 5 Discussion

Previous studies in the HRM-Performance equation were concentrated in developed and emerging economies. The objective of this article was to investigate the underlining mechanism through which HRM System affects the performance of public service organizations in Ethiopia, one of the developing countries. Drawing on AMO and social exchange theory, the present study hypothesized to examine the effect of HRM Systems on employee engagement and perceived organizational performance and examining the mediating effect of employee engagement on the HRM-Performance research. In this respect, all the hypotheses were accepted.

As it was hypothesized, it was found that HRM has a positive and significant effect on organizational performance. This result is consistent with the previous empirical findings (Wright et al., 2005*;* Combs et al., 2006*;* Ko and Smith, 2013*;* Kehoe and Wright, 2013*;* Ko and Walter-Smith, 2013*;* Vermeeren, 2014). It was argued by Appelbaum et al. (2000) that organizational success and comparative advantage don't just come from strategy, product and service alone, rather through an organization's ability to efficiently and effectively oversee its human resources. The investment in people and valuing its employees enhances the performance of government organizations (Kim, 2005). In Ethiopia, public service organizations are government organs which provide and plays a key role in delivering the basic service to its citizens such as education, health, transport, telecommunication, electricity, water, and others. Such basic services are provided by the employees and so it is necessary to develop sound HRM in order to bring better performance levels to their customers measured in terms of productivity, quality, satisfaction, fairness, efficiency, and equity. In today's competitive environment, modern organizations are working day and night to have a productive workforce. Thus, pressure on the government and public service organization leaders can be managed if they strategically consider employees as an asset and ally for their mission accomplishment (*Kim, 005*). Interestingly, Ahmed and Schroedar (2003) believed that sophistication in technology and innovation in manufacturing practices alone do little effect to improve performance unless there is a presence of sound HRM practice that enhances organizational performance.

Finding of this study also established a positive relationship between HRM and Employee Engagement, indicating that employees become engaged when they believe that they are treated, valued and trusted (Saks, 2006; Rich et al., 2010; Alfes et al., 2013). This is in line with the AMO and SET that the degree of engagement level highly depends on the reaction from their organization's practices. Drawing on the social exchange theory, if organizations invest and treat their employees as strategic partners and assets, employees become happier and engaged. As to AMO theory, skill-enhancing bundles improve the ability, knowledge, and attitude of employees and this helps employees to be more engaged with their work; motivation enhancing bundles improves the willingness and degree of effort of employees to exert on their work and this directly may enhance the engagement level of employees. Largely, the finding of the study established that HRM is a strong predictor of employee engagement.

Similarly, the present study found a positive and significant link between employee engagement and performance, which is consistent with prior studies (Harter et al., 2002; Markos and Sridevi, 2010*;* Sundaray, 2011; Harter et al 2002; Rees et al., 2013*;* Sibanda et al., 2014). The main point here is that employee engagement is a strong predictor of performance of public service organizations because an engaged workforce is happy, motivated and hence can boost organizational performance (Rees et al., 2013). And also Sundaray (2011) posited out employee engagement as a decisive factor for organizational effectiveness (productivity, profits, quality, and customer satisfaction). That means an engaged workforce can fully contribute to performance with happiness, dedication, and motivation.

Regarding the mediation effect, the finding of the study ensured partial mediation. Specifically, it was found that as hypothesized, employee engagement partially mediated the relationship between HRM and Organizational Performance. The finding is consistent with previous findings (Saks, 2006*;* Rich et al., 2010*;* Sundaray, 2011*;* Truss et al., 2013*;* Alfes et al., 2013*;* Andrew and Sofian, 2012*;* Mudhi et al, 2016). The implication is that a sound and effective implementation of HR practices as a system enables to create an engaged workforce and in return enhances better organizational performance. It was identified by Sundaray (2011) that most of the HR practices such as training and development, performance appraisal, career development, recruitment, job design, compensation, leadership, fair treatment, safety, and health, communication, among others, leads to employee engagement and in return improves organizational effectiveness. The implication is that HRM is an important predictor of both employee engagement and organizational success. The finding of this study is consistent with the black-box mechanism in that system of HRM practices first affects employee engagement and then in return affects operational performance outcomes such as productivity, customer satisfaction, and quality and so on. And also the issue of black-box in the relationship between HRM and Organizational Performance was treated through employee engagement, suggesting that the result of this study is consistent with the AMO theory.

Finally, this study also examined the differing effect of the six HR practices separately on both employee engagement and organizational performance. The finding of the study supported that all six HR practices have a significant and positive effect on both employee engagement and organizational performance in the public service. From the individual HR practices, autonomy was found as an important driver in both employee engagement and organizational performance. This means that if employees have given the freedom and possibility of involving in decision making, offering their comments and providing feedback in the operation of the organizations, then it will enhance the engagement level of employees. The implication is that the HRM in their system form and individually have an effect on the performance of organizations.

## 6 Conclusion

Understanding the nexus between HRM and Organizational performance is one of the continuing goals of HR scholars (Jackson et al., 2014; Vermeeren, 2014). This article is among the few studies in the HRM-Performance debate using mediation model in public service organizations and developing country's context. Drawing on AMO and social exchange theory, the present study hypothesized to investigate the effect of HRM Systems on employee engagement and perceived organizational performance and testing the intervening effect of employee engagement on the link between HRM and organizational performance. The findings of the study demonstrated a positive and significant relationship between HRM Systems, employee engagement and perceived organizational performance. It was also found that employee engagement partially mediate the HRM-Performance Linkage. This implies that investment in the human resource and building sound HRM system will help organizations to produce an engaged workforce, which in return improves the performance of organizations. Conclusively, this article contributes to the body of knowledge in the HRM-Performance debate in the developing country's public service organizations and supported the universal perspective and mediation approach.

## 7 Theoretical implications

The finding of this study offers strong support for the direct and indirect effect of HRM system on the performance of public service organizations in the Ethiopian setting. This study contributed to the HRM literature that attitudinal variables (employee engagement) can mediate the mechanism through which HRM-related organizational performance. In the HRM literature, there are two paths (direct and indirect) of research to link the HRM and organizational performance (Darwish, 2013). Recently, due to their inconclusive results, studies focused on the indirect effect of HRM on organizational performance through a mediation mechanism to understand the HRM-performance nexus. Following this trend, this study empirically tested the intervening role of employee engagement in the foregoing relationship. This implies that appropriate implementation of HRM practices helps employees to develop a better perception of their organization which in turn enable them to be engaged in their job and organization. And also, an engaged worker becomes satisfied, dedicated and immersed which leads to enhanced performance.

This study also contributes to the HRM research by supporting the systems approach which demonstrates that the whole is greater than its components. In this respect, individual HR practices are effective by combining complementary HR practices together instead of the individual practices separately. According to Lepak et al. (2006)*,* the system's perspective is very critical to scrutinize the effect of HR practices on organizational benefits. The essence of the systems perspective is that if the critical elements of the HRM activities are well organized and coordinated, then the effect on the performance of an organization is more significant when the HR practices are implemented as a system/bundle rather than individual practices as in isolation. That means the effect of the system/bundles of HRM on both individual and organizational performance is much greater than the individual HR practices alone (Huselid, 1995; MaccDuffie, 1995; Datta et al., 2005; Lepak et al., 2006; Combs et al., 2006; Jiang et al., 2012).

It also supports the AMO framework that HR practices can be classified into Ability, Motivation, and Opportunity so that they can contribute to performance. Boxall and Purcell (2008) argued that organizational performance is a result of ability (good recruitment and training system), motivation (fair compensation and appraisal system and opportunity (degree of autonomy and involvement to participate in organizational matters). The fundamental notion of AMO model is that employees can be engaged and productive in themselves as well as their organization's performance when they are equipped with the necessary skill and ability; when they are motivated and provided the opportunity to involve in organizational matters (Appelaum et al, 2000). Moreover, the finding of the present study is aligned with the social exchange theory. In this nation, proper treatment and management of the employees at the workplace are encouraging for them. Such appropriate actions of their organizations positively reacts to their organization by being engaged and motivated and contribute to their organizations to enhance the performance of the organizations.

## 8 Practical implications

The study has practical implications. It was found that HRM is a strong predictor of both employee engagement and organizational performance. This implies that if there is sound HRM and there is proper implementation of the HR practices, then employees will have the appropriate skill and knowledge to perform their work; create proper motivation and have the opportunity to operate their work. This all will help them to be engaged in their work. If there is an engaged workforce in an organization, then, the performance of organizations will be improved. Armstrong and Taylor (2014) noted that fully engaged employees are happy and more productive, unlike employees who develop poor relationships with their supervisors leading to disengagement. It is recognized that if HRM is not properly administered, employees fail to fully engage in their work and lead to lower organizational performance (Markos and Sridevi, 2010). So, public service organization leadership including the immediate supervisors should invest much time, effort and attention to the management of HR and implement a sound HRM System at the workplace so that employees will have a positive reaction towards their organizations. As a result, they will help to build an engaged workforce who is satisfied, motivated and committed to providing the expected service in the government organizations with efficiency, effectiveness, and fairness.

## 9 Limitation and future research direction

This study is subject to some limitations. The first limitation is related to the duration of the study. This study is cross-sectional in nature where data needed for all the study constructs were collected in one period in time. Cross-sectional studies didn't provide the opportunity to see the cause-effect between constructs. Thus, it is better to conduct longitudinal studies in the future to conclude sound and robust causality. Second, this study collects data from the same sources for all the constructs (HRM, employee engagement and organizational performance) which may lead to common method variance. However, the present study collected data from different types of employees from different public service organizations in various hierarchies. To minimize the common method variance, the study followed the suggestion of Podsakoff et al. (2012) such as ensuring confidentiality, psychological separation of measurement scales, and application of simple and easy language in the survey questionnaire. Besides, this study confirms, using Herman's Single Factor, that common method variance is not a serious problem. Nevertheless, in the future, researchers are suggested to collect data from different sources and raters to avoid common method variance.

The third limitation of the study was measuring organizational performance subjectively. It can be measured through both objective as well as subjective measures. However, an objective measure of organizational performance was difficult to get in public government organizations. Other scholars have also relied on subjective evaluation of organizational performance (Berewer and Selden, 2000; Kim, 2005; Sahin, 2010; Ko and Smith, 2013; Vermeeren, 2014; Riaz, 2015; Kula and Sahin, 2016; Emhan et al., 2016*)*. Fourth, the study was based on the individual level of analysis in examining the HRM-Performance equation. Although it is appropriate to evaluate the link in this way, recent researches calls are suggesting to conduct multi-level analysis in examining the HRM-Performance debate. So, future researchers are recommended to conduct both organizational and individual levels together in the HRM-Performance Linkage. Fifth, this study only considers HRM as an antecedent of employee engagement. Despite the fact that the major organizational factor that affects employee engagement is HRM practices, different factors, for example, leadership, and working climate can be considered in the future. This is because there are many factors that enhance employee engagement which in return affect organizational outcomes. Finally, to avoid the inter-industry effect, this study focused on one sector and it didn't take into account the cultural influence in the link between HRM and Organizational Performance. In any case, it is imperative to consider and comprehend the cross-cultural issues in measuring employee engagement (Truss et al., 2013). In strengthening the above issue, Hofstede (2011) argued that different people behave differently due to the existence of national culture. The practice of HRM, employee engagement and Performance may be affected by the presence of national culture. Thus, future researchers should consider the culture and different contexts in measuring the contribution of HRM to the performance of organizations.

## Declarations

### Author contribution statement

Assefa Tsegay Tensay, Manjit Singh: Conceived and designed the experiments; Performed the experiments; Analyzed and interpreted the data; Wrote the paper.

### Funding statement

This research did not receive any specific grant from funding agencies in the public, commercial, or not-for-profit sectors.

### Competing interest statement

The authors declare no conflict of interest.

### Additional information

No additional information is available for this paper.
